# In Vitro Screening Studies on Eight Commercial Essential Oils-Derived Compounds to Identify Promising Natural Agents for the Prevention of Osteoporosis

**DOI:** 10.3390/biomedicines11041095

**Published:** 2023-04-04

**Authors:** Marta Trzaskowska, Vladyslav Vivcharenko, Paulina Kazimierczak, Agata Wolczyk, Agata Przekora

**Affiliations:** Independent Unit of Tissue Engineering and Regenerative Medicine, Medical University of Lublin, Chodzki 1, 20-093 Lublin, Poland

**Keywords:** osteoblasts, mesenchymal stem cells, cytotoxicity, cell proliferation, terpenes

## Abstract

Over the years, essential oils (EOs) and their compounds have gained growing interest due to their anti-inflammatory, antimicrobial, antioxidant, and immunomodulatory properties. The aim of this study was to evaluate the effect of eight commercially available EO-derived compounds ((R)-(+)-limonene, (S)-(−)-limonene, sabinene, carvacrol, thymol, alpha-pinene (α-pinene), beta-pinene (β-pinene), and cinnamaldehyde) on the bone formation process in vitro to select the most promising natural agents that could potentially be used in the prevention or treatment of osteoporosis. Within this study, evaluation of cytotoxicity, cell proliferation, and osteogenic differentiation was performed with the use of mouse primary calvarial preosteoblasts (MC3T3-E1). Moreover, extracellular matrix (ECM) mineralization was determined using MC3T3-E1 cells and dog adipose tissue-derived mesenchymal stem cells (ADSCs). The two highest non-toxic concentrations of each of the compounds were selected and used for testing other activities. The conducted study showed that cinnamaldehyde, thymol, and (R)-(+)-limonene significantly stimulated cell proliferation. In the case of cinnamaldehyde, the doubling time (DT) for MC3T3-E1 cells was significantly shortened to approx. 27 h compared to the control cells (DT = 38 h). In turn, cinnamaldehyde, carvacrol, (R)-(+)-limonene, (S)-(−)-limonene, sabinene, and α-pinene exhibited positive effects on either the synthesis of bone ECM or/and mineral deposition in ECM of the cells. Based on the conducted research, it can be assumed that cinnamaldehyde and (R)-(+)-limonene are the most promising among all tested EO-derived compounds and can be selected for further detailed research in order to confirm their biomedical potential in the chemoprevention or treatment of osteoporosis since they not only accelerated the proliferation of preosteoblasts, but also significantly enhanced osteocalcin (OC) synthesis by preosteoblasts (the OC level was approx. 1100–1200 ng/mg compared to approx. 650 ng/mg in control cells) and ECM calcification of both preosteoblasts and mesenchymal stem cells. Importantly, cinnamaldehyde treatment led to a three-fold increase in the mineral deposition in ADSCs, whereas (R)-(+)-limonene caused a two-fold increase in the ECM mineralization of both MC3T3-E1 cells and ADSCs.

## 1. Introduction

The skeletal system is a body support that participates in the movement of the whole body and protects the internal organs. Apart from structural support, it is a reservoir of minerals and even performs an endocrine activity [1]. In order to function efficiently, the skeleton undergoes constant reconstruction, in which bone-forming osteoblasts and bone-resorbing osteoclasts are involved. Imbalance during bone remodeling processes can lead to a loss of homeostasis and the subsequent development of metabolic diseases [2]. The most common skeleton disease is osteoporosis, which is characterized by a reduction in bone mass and microarchitecture defects, leading to the loss of bone strength and a high risk of fractures [3]. Risk factors for osteoporosis are age, female gender, Caucasian race, low level of physical activity, and calcium and vitamin D deficiency in the diet [4]. Currently, the pharmaceutical treatment of osteoporosis is based on two strategies: anti-resorptive and anabolic medications. The action of anti-resorptive agents, such as bisphosphonates, estrogens, selective estrogen receptor modulators (SERM), or denosumab is based on the disruption of osteoclast function. In turn, anabolic drugs, e.g., parathyroid hormone (PTH) analogs and romosozumab, stimulate the activity of osteoblasts [5,6]. However, these therapies have a number of side effects. The use of bisphosphonates may result in osteonecrosis of the jaw [7], while long-term estrogen therapy increases the risk of breast cancer and stroke [6]. Hormone replacement therapy may contribute to an increased risk of breast cancer due to the genotoxic effects of estrogen metabolites. Moreover, estrogens stimulate cell proliferation, which indirectly increases the likelihood of DNA mutations [8,9].

Natural compounds may be a promising alternative in the prevention of osteoporosis, due to their safety and low cost of treatment [10,11]. Polyphenols and anthocyanins, commonly found in fruits and vegetables, may decrease the risk of developing osteoporosis thanks to their antioxidant activity. By eliminating the negative effects of reactive oxygen species (ROS), these antioxidants contribute to the stimulation of osteoblast differentiation and mineralization and reduce the differentiation and activity of osteoclasts [12]. Among polyphenols, phytoestrogens belong to a special group in the context of the prevention of osteoporosis in women. Studies have shown that consuming soybean phytoestrogens can protect against bone loss. They exhibit estrogen-like activity, but they act in a more sustainable way, which is especially important in the case of tissues that are hormone-sensitive. Phytoestrogens may act directly through estrogen receptors or indirectly, by regulating the activity of other hormones involved in the bone remodeling process or reducing the synthesis of pro-inflammatory cytokines [5,13,14].

Safe alternatives to the chemoprevention and treatment of osteoporosis are plant-derived essential oils (EOs) [15]. EOs are multicomponent mixtures that contain hundreds of compounds, such as monoterpenes (sabinene, alpha-pinene, beta-pinene, limonene), terpenoids (carvacrol, thymol, menthol, geraniol), and phenylpropanoids (cinnamaldehyde, eugenol, safrole) [15,16,17,18]. EOs have many beneficial biological properties, e.g., analgesic [19], antibacterial [17], antifungal [20], anti-inflammatory, and antioxidant [21]. In addition, the lipophilic nature of EOs affects an easy penetration of cell membranes, effectively regulating cellular functions [22]. Moreover, some reports showed that EO-derived compounds exerted an influence on bone regeneration by inhibiting osteoclast differentiation and stimulating the apoptosis process in mature osteoclasts [23,24,25,26]. In vitro studies showed that curcumol, a major component of *Rhizoma Curcumae* essential oil, at the concentration of 40 and 160 μM, inhibited receptor activator of nuclear factor-kappa B ligand (RANKL)-induced osteoclast differentiation from murine macrophages (RAW264.7) and bone marrow-derived macrophages (BMM) [23]. Thymol, as one of the main oils derived from thyme, at a concentration of 10–40 μM, also exhibited similar activity against RAW264 and BMM macrophages without toxic effect. Oral administration of thymol (25 mg/kg and 100 mg/kg) significantly reduced lipopolysaccharides (LPS)-stimulated inflammatory bone loss in mice [24]. Deepak et al. showed that carvacrol at the concentration of 3–15 µg/mL reduced RANKL-induced osteoclastogenesis, and, at the concentration of 15 µg/mL, also reduced the LPS-induced process. Moreover, it stimulated apoptosis in mature osteoclasts in vitro [26]. In other studies, it was shown that some terpenes (e.g., limonene, alpha-pinene) stimulated osteoblast proliferation and differentiation, which is similar to anabolic drug action used in the treatment of osteoporosis [22,27,28,29]. In vitro studies revealed that limonene induced osteoblastic differentiation of the murine myoblasts (C2C12) (concentration: 2.5 µM) and murine preosteoblasts (MC3T3-E1) (concentration: 0.01–1 μM) [27]. Moreover, limonene (0.01–1 μM) decreased methylglyoxal-induced endoplasmic reticulum (ER) stress and autophagy in the MC3T3-E1 cell line [28]. Another monoterpene, α-pinene (50 μM), also stimulated the differentiation and mineralization process of osteoblasts in the MC3T3-E1 cells [29]. There are also reports indicating that cinnamaldehyde prevented osteoporosis. Wu et al. showed an increase in the number of osteoblasts and a decrease in the number of osteoclasts in the distal femur of rats that had cinnamaldehyde administered by gavage [30]. Thus, the therapeutic potential of EOs may be used as an alternative method for bone diseases care.

Since the effect of specific EO-derived compounds on osteoblast behavior and the bone formation process is not well studied, the aim of this screening research was to evaluate osteoblast proliferation and osteogenic differentiation of osteoblasts and mesenchymal stem cells in response to stimulation with eight commercially available compounds: (R)-(+)-limonene, (S)-(−)-limonene, sabinene, carvacrol, thymol, alpha-pinene (α-pinene), beta-pinene (β-pinene), and cinnamaldehyde. It is worth noting that according to the available literature, there are no papers showing a comparative and comprehensive assessment of the osteogenic potential of the different EO-derived compounds. Within this study, evaluation of cytotoxicity, cell proliferation, osteogenic differentiation, and extracellular matrix (ECM) mineralization with the use of mouse primary calvarial preosteoblasts (MC3T3-E1) and dog adipose tissue-derived mesenchymal stem cells (ADSCs) was carried out. Performed studies allowed the selection of the most promising natural-derived compounds that may be used in the prevention of osteoporosis. The occurrence and structural formulas of the tested EO-derived compounds are presented in Table 1.

## 2. Materials and Methods

### 2.1. EO-Derived Compounds

Eight commercially available EO-derived compounds were tested. The following compounds were obtained from Sigma-Aldrich Chemicals (Warsaw, Poland): cinnamaldehyde (*trans*-3-phenyl-2-propenal, MW = 132.16 g/mol), carvacrol (5-isopropyl-2-methylphenol, MW = 150.22 g/mol), thymol (5-methyl-2-isopropylphenol, MW = 150.22 g/mol), (*R*)-(+)-Limonene ((*R*)-4-isopropenyl-1-methyl-1-cyclohexene, MW = 136.23 g/mol), (*S*)-(−)-limonene ((*S*)-4-isopropenyl-1-methyl cyclohexene, MW = 136.23 g/mol), sabinene (4-methylidene-1-propan-2-ylbicyclo [3.1.0]hexane, MW = 136.23 g/mol), α-pinene (2,6,6-Trimethylbicyclo [3.1.1]hept-2-ene, MW = 136.23 g/mol), and β-pinene (6,6-Dimethyl-2-methylenebicyclo [3.1.1]heptane, MW = 136.23 g/mol). For testing, starting stock solutions at 100 mg/mL were prepared in dimethyl sulfoxide (DMSO) (Sigma-Aldrich Chemicals, Warsaw, Poland).

### 2.2. Cell Culture Experiments

#### 2.2.1. Cells and Cell Cultures

The screening cytotoxicity test, cell proliferation assay, and osteogenic differentiation tests were carried out using a mouse primary calvarial preosteoblast cell line (MC3T3-E1 Subclone 4, CRL-2593, ATCC-LGC standards, Teddington, UK). MC3T3-E1 cells were cultured in Alpha Minimum Essential Medium (GIBCO, Life Technologies, Carlsbad, CA, USA) with 10% fetal bovine serum (FBS, Pan-Biotech GmbH, Aidenbach, Bavaria, Germany) and antibiotics (100 U/mL penicillin, 0.1 mg/mL streptomycin) (Sigma-Aldrich Chemicals, Warsaw, Poland). MC3T3-E1 cells were maintained at 37 °C in a humidified atmosphere of 5% CO_2_ and 95% air. In turn, evaluation of extracellular matrix (ECM) mineralization was performed using MC3T3-E1 cells and adipose tissue-derived mesenchymal stem cells (ADSCs) isolated from dog adipose tissue (fat tissue was collected during the sterilization procedure of a female dog after obtaining written informed consent from the dog owner). Isolation of ADSCs was performed according to the procedure described elsewhere [41,42]. To confirm the successful isolation of dog ADSCs, immunofluorescent staining of specific ADSC markers (integrin β1/CD29 and CD90/Thy1) was performed and shown in our previous study [42]. ADSCs were cultured in a 1:1 mixture of DMEM/Ham’s F12 medium without phenol red, containing 2.5 mM L-glutamine (Sigma-Aldrich Chemicals, Warsaw, Poland), 10 ng/mL rhFGF, 5 ng/mL rhEGF (Pan-Biotech GmbH, Aidenbach, Bavaria, Germany), 10% FBS, and antibiotics (100 U/mL penicillin, 0.1 mg/mL streptomycin). ADSCs were maintained at 37 °C in a humidified atmosphere of 5% CO_2_ and 95% air.

#### 2.2.2. Cytotoxicity Screening

For cytotoxicity screening, serial two-fold dilutions (ranging between 1 and 1000 μg/mL) of the essential oil-derived compounds in cell medium were performed. First, 100 μL of the MC3T3-E1 cells at a concentration of 2 × 10^5^ cells/mL were seeded in 96-multiwell plates. After 24 h incubation at 37 °C, the culture medium was replaced with 100 μL of different concentrations of tested compounds. To exclude potential cytotoxicity of the solvent (DMSO) of EO-derived compounds, DMSO solutions at the range of the concentrations used in the cytotoxicity screening assay were tested in parallel. After 24 h exposure of cells to the essential oil-derived compounds, cell viability was determined by MTT (3-(4,5-dimethylthiazol-2-yl)-2,5-diphenyltetrazolium bromide) assay (Sigma-Aldrich Chemicals, Warsaw, Poland). EO concentrations were discarded from the 96-multiwell plates and 100 μL of freshly prepared MTT solution (1 mg/mL) in culture medium was added to the wells. After 3 h of incubation at 37 °C, 100 µL of 10% sodium dodecyl sulfate (SDS) solution (Sigma-Aldrich Chemicals, Warsaw, Poland) in 0.01 M HCl (Avantor Performance Materials, Gliwice, Poland) was added. The 96-multiwell plates were placed in the incubator for 12 h and then the absorbance value was measured at a wavelength of 570 nm using a microplate reader (BioTek Synergy H4 Hybrid Microplate Reader, Winooski, VT, USA). The results obtained from the colorimetric assay were shown as the percentage of absorbance value obtained with the negative cytotoxicity control (cells cultured in a medium without essential oils), revealing 100% viability. After cytotoxicity screening, the two highest concentrations that did not cause a statistically significant decrease in cell viability (cell viability ≥ 95%) of each essential oil-derived compound were subjected to further cell experiments.

#### 2.2.3. Cell Proliferation Assessment

MC3T3-E1 cells were seeded in 96-multiwell plates in 100 μL of complete culture medium at a concentration of 0.5 × 10^5^ cells/mL. After 24 h incubation at 37 °C, the culture medium was replaced with 100 μL of the highest non-toxic concentrations of each essential oil-derived compound. After 24 h and 72 h exposure of cells to the compounds, MC3T3-E1 cells were lysed, and the exact cell number was calculated based on the release of total LDH from the cell population using the Lactate Dehydrogenase Activity (LDH) Assay Kit (Sigma-Aldrich Chemicals, Warsaw, Poland). In this test, unlike a typical LDH cytotoxicity assay, the higher the content of released LDH observed, the greater number of cells present in the population. The experiment was performed according to the manufacturer’s protocol. The cell concentration after 24 h and 72 h cultures was estimated from the calibration curve made for the known number of lysed MC3T3-E1 cells. The doubling time (DT) for the cells was calculated using Doubling Time Computing software version 3.1.0.

#### 2.2.4. Evaluation of Osteogenic Differentiation

MC3T3-E1 cells were seeded in 48-multiwell plates in 500 μL of appropriate complete culture medium at a concentration of 1 × 10^5^ cells/mL. After 24 h incubation at 37 °C, the culture medium was replaced with 500 μL of osteogenic medium containing essential oil-derived compounds. The osteogenic medium was prepared by adding the following supplements to a complete culture medium: 0.05 mg/mL ascorbic acid and 10 mM β-glycerophosphate (Sigma-Aldrich Chemicals, Warsaw, Poland). The experiment was performed for 21 days, and on every 3rd day, half of the osteogenic medium containing the appropriate concentration of essential oil-derived compound was replaced with a fresh portion. After 8 days of experiment, type I collagen (Col I) and bone alkaline phosphatase (bALP) were quantified in cell lysates using appropriate enzyme-linked immunosorbent assays (ELISAs) specific to mouse. Osteocalcin (OC) level was determined after 21 days. The time intervals of marker determination were selected taking into account the periods of their increased synthesis by cells during the differentiation process (Col I, bALP—early markers, CO—late marker) [43]. For this purpose, the culture medium was removed, a solution of protease inhibitor cocktail (PIC, Sigma-Aldrich Chemicals, Warsaw, Poland) in phosphate-buffered saline (PBS) (Sigma-Aldrich Chemicals, Warsaw, Poland) was prepared, maintaining a dilution ratio of 1:100, and then 500 µL of PIC solution was added to each well. The cells were lysed by two freeze–thaw cycles and sonication for 30 s at 30% amplitude, as described previously [44]. Commercially available ELISAs were conducted using cell lysates according to the manufacturer’s protocols (EIAab ELISAs kit, Wuhan, China). Additionally, the total cellular proteins in cell lysates were determined by using a Pierce BCA Protein Assay (Thermo Fisher Scientific, Waltham, MA, USA). Results obtained from ELISAs were expressed as ng of osteogenic marker per mg of total cellular proteins.

#### 2.2.5. Evaluation of ECM Mineralization

Alizarin red S (ARS) staining is one of the methods of detecting and determining the level of calcium compounds in vitro and monitoring skeletal mineralization in vivo. ARS has a high affinity for binding to calcium ions, thus forming a complex with a color similar to orange and red. It is often used to assess the osteogenic potential of mesenchymal stem cells and osteoblasts and in screening studies for compounds with potential pro-osteogenic activity. It allows for both qualitative (microscopic imaging) and quantitative (spectrophotometer measurements) evaluations of ECM mineralization [45,46]. MC3T3-E1 cells and ADSCs were seeded in 48-multiwell plates in 500 μL of appropriate complete culture medium at a concentration of 1 × 10^5^ cells/mL. After 24 h incubation at 37 °C, the culture medium was replaced with 500 μL of osteogenic medium containing essential oil-derived compounds, and the cells were cultured as described above. After a 21-day exposure of MC3T3-E1 cells and ADSCs to the osteogenic medium supplemented with tested compounds, qualitative and quantitative evaluation of the mineral deposition in ECM was conducted by Alizarin Red S staining, according to the procedure described in our previous study [47]. In brief, cells were fixed with 3.7% paraformaldehyde, rinsed twice with PBS, and stained for 15 min at 37 °C with a 2% (*w*/*v*) ARS (all reagents were purchased from Sigma-Aldrich Chemicals, Warsaw, Poland) solution prepared in deionized water. Then, the staining solution was discarded and the cells were washed five times with PBS without calcium and magnesium ions to remove unbounded dye. The mineral deposits in the ECM were visualized by a stereoscopic microscope (Olympus SZ61TR, Olympus Polska Sp. z o. o., Warsaw, Poland). After visualization, the same samples were subjected to quantitative evaluation of the mineral deposits in the ECM according to the procedure described earlier [47]. The calcium-bound ARS was dissolved by using 10% acetic acid (Sigma-Aldrich Chemicals, Warsaw, Poland). Next, mineralized ECM along with calcium-bound ARS were subjected to heating at 85 °C for 10 min. Afterwards, the samples were centrifuged and then the obtained supernatants were neutralized with 10% ammonium hydroxide (Avantor Performance Materials, Gliwice, Poland). The color of the resultant solution is directly proportional to the calcium deposited in the cell culture. Thus, the greater the OD values detected, the more mineral was present in the culture. The extracted calcium-bound ARS solution was used for colorimetric quantification at 405 nm using a microplate reader (BioTek Synergy H4 Hybrid Microplate Reader, Winooski, VT, USA).

### 2.3. Statistical Analysis

All tests were performed in triplicate. Statistical analysis was performed using an unpaired *t*-test and one-way ANOVA, followed by Tukey’s test (GraphPad Prism 8.0.0 Software, GraphPad Software Inc., California, CA, USA). The significance level was considered at *p* < 0.05. The results were expressed as the mean values ± standard deviation (SD).

## 3. Results and Discussion

### 3.1. Cytotoxicity Screening

In vitro cytotoxicity screening of the wide range of selected commercially available EOs was conducted on a mouse preosteoblast cell line (MC3T3-E1 Sublone 4) using an MTT assay. The conducted study allowed the selection of the two highest non-toxic concentrations of each compound, which were used in further tests. Concentrations ranging from 1 to 1000 µg/mL of each compound were assessed. Based on the performed test, the highest non-toxic concentrations ranged from 7.50 to 300 µg/mL, depending on the compound (Table 2). Cinnamaldehyde showed the highest cytotoxicity among all the tested compounds since the highest non-toxic concentration was equal to 15.00 µg/mL. In turn, thymol and carvacrol did not adversely affect cell viability at concentrations up to 37.50 μg/mL. (R)-(+)-limonene, (S)-(−)-limonene, and sabinene were non-toxic at concentrations up to 62.50 μg/mL, whereas α-pinene and β-pinene showed very low toxicity. Both mentioned compounds did not reveal any toxic effect on MC3T3-E1 cells at as high a concentration as 300 µg/mL. Table 2 presents the non-toxic concentrations of the EO-derived compounds selected for further experiments. Higher concentrations than those shown in the table were cytotoxic and caused a significant reduction in cell viability compared to the control sample. Based on the obtained results, it may be concluded that the tested EO-derived compounds differ significantly in the highest non-toxic concentrations. Some published scientific reports indicated that the cytotoxic effect of essential oils is a result of the depolarization of mitochondrial membranes, which, in turn, significantly increases their permeability [48]. In addition, large differences in the selected non-toxic concentrations may be closely related to the chemical structure of the compounds, as monoterpenes (limonenes, pinenes, sabinene) showed the lowest cytotoxicity, terpenoids (thymol, carvacrol) were medium, and phenylpropanoid (cinnamaldehyde) showed the highest cytotoxicity. In the next step of the study, the two highest non-toxic concentrations of each of the tested compounds were subjected to evaluation of their effects on osteoblast proliferation, osteogenic differentiation, and ECM mineralization in vitro.

### 3.2. Cell Proliferation Assessment

**Figure 1:**
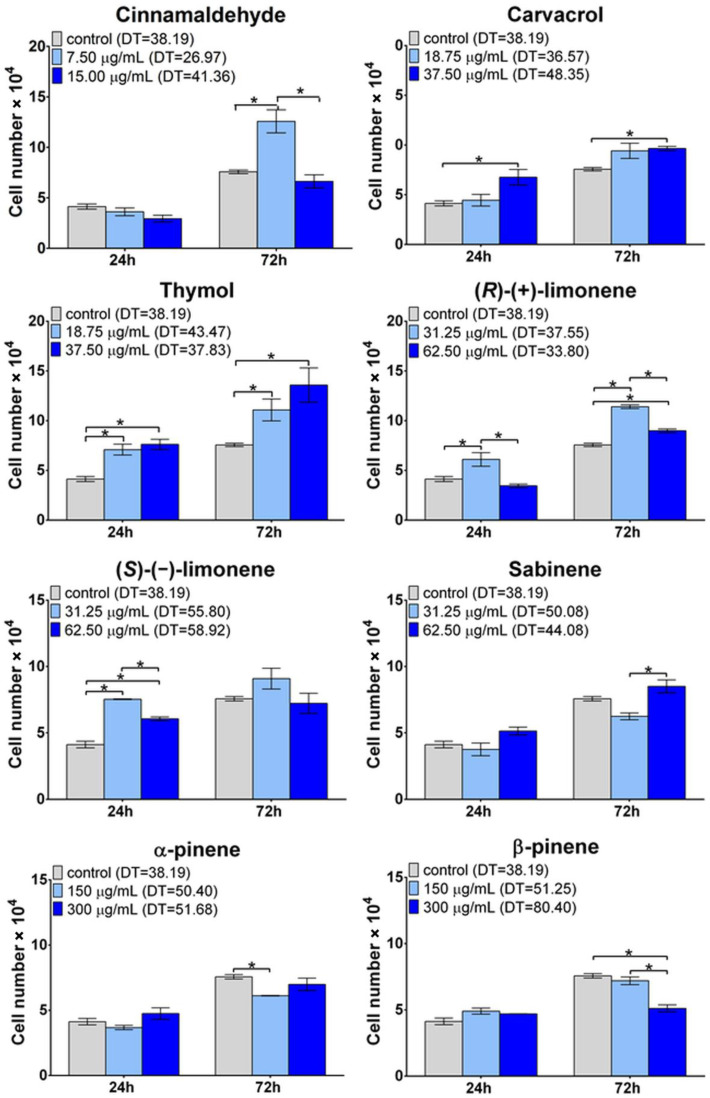
Influence of EO-derived compounds on mouse primary calvarial preosteoblast (MC3T3-E1 Subclone 4) proliferation in vitro. The total cell number was calculated using the Lactate Dehydrogenase Activity (LDH) Assay Kit (DT, doubling time in hours; * statistically significant results between indicated groups, *n* = 3, *p* < 0.05, one-way ANOVA followed by Tukey’s test).

A rapid increase in the number of mature osteoblasts and their high activity in bone matrix production are key factors in bone remodeling and the proper regeneration of bone tissue [49]. Intense osteoblast proliferation is very important since it ensures optimal cell mass for an efficient osteogenic differentiation process [50]. In this study, the cell number in the population was assessed after 24- and 72-h exposure of the osteoblasts to the selected non-toxic concentrations of the EO-derived compounds (Figure 1). Among all the tested EO-derived compounds, cinnamaldehyde at the concentration of 7.50 µg/mL, thymol at the concentration of 37.50 µg/mL, and (R)-(+)-limonene at the concentration of 31.25 µg/mL were the most favorable to cell proliferation. Moreover, the doubling time (DT defined as a period of time in hours needed to double the cell population) calculated for the cells cultured with cinnamaldehyde (DT = 26.97 h) was meaningfully shorter than the DT of the control cells cultured without any compounds (DT = 38.19 h), proving that cinnamaldehyde significantly enhanced cell proliferation in vitro. In the case of carvacrol, a statistically significant increase in the cell number was observed only at 37.50 µg/mL after both time intervals compared to the control. However, only a slight enhancement of cell proliferation was noted. (S)-(−)-limonene significantly increased the osteoblast proliferation at concentrations of 31.25 and 62.50 µg/mL after 24 h. However, after 3 days, the cell number was comparable to the control. In turn, sabinene, α-pinene, and β-pinene did not reveal a significant beneficial effect on cell proliferation; moreover, for some samples, a decrease in cell number was observed. The decrease in the number of cells over time may indicate that the osteoblasts of the MC3T3-E1 line have entered a stationary phase and stopped cell division. To sum up, a positive impact on cell proliferation was observed for cinnamaldehyde, carvacrol, thymol, (R)-(+)-limonene, and (S)-(−)-limonene. However, significantly enhanced cell proliferation with scientific and biomedical importance was noted only after treatment with cinnamaldehyde, thymol, and (R)-(+)-limonene. Importantly, according to previous scientific research, thymol and carvacrol revealed a promoting impact on the proliferation of fibroblasts, keratinocytes, and adipose tissue-derived stem cells, which is consistent with this study [51].

### 3.3. Evaluation of Osteogenic Differentiation

**Figure 2:**
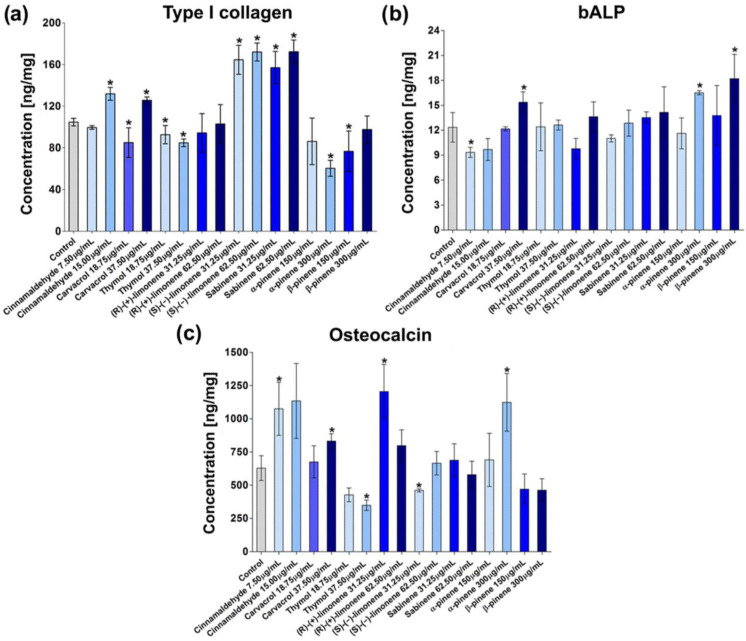
The level of osteogenic markers produced by mouse primary calvarial preosteoblasts (MC3T3-E1 Subclone 4) in response to essential oil-derived compounds: (**a**) type I collagen, (**b**) bone alkaline phosphatase (bALP), and (**c**) osteocalcin (* statistically significant results compared to control, *n* = 3, *p* < 0.05, unpaired *t*-test).

The bone matrix is one of the main components of bone tissue and is mainly built of type I collagen (Col I) (90%) and non-collagenous proteins (10%) such as osteonectin, bone sialoproteins, osteocalcin, and various proteoglycans [52]. According to the studies, bone toughness is mainly determined by the collagen matrix, while bone strength and stiffness depend on the mineral phase. Changes in bone toughness were observed after bone collagen damage using ionizing radiation [53]. Thus, induction of collagen synthesis plays an important role during osteoporosis treatment. In this study, the effect of EO-derived compounds on collagen synthesis by MC3T3-E1 cells after an 8-day culture was studied (Figure 2a). Among all the tested EO-derived compounds, cinnamaldehyde at the concentration of 15 µg/mL, carvacrol at the concentration of 37.50 µg/mL, (S)-(−)-limonene at the concentration of 31.25 µg/mL and 62.50 µg/mL, and sabinene at the concentration of 31.25 µg/mL and 62.50 µg/mL significantly increased type I collagen (Col I) production in MC3T3-E1 cells.

The regulation of collagen synthesis seems to be a promising approach for bone fragility treatment [54]. Taking into account the revealed stimulative effects of cinnamaldehyde, carvacrol, (S)-(−)-limonene, and sabinene on the synthesis of the main bone matrix component, it may be concluded that the mentioned compounds derived from EOs may reduce bone brittleness. The obtained results are partially consistent with the reports presented by other authors who confirmed the positive impact of limonene and cinnamaldehyde on collagen synthesis [28,30].

Bone alkaline phosphatase (bALP) is the typical osteogenic marker that promotes mineralization and suppresses osteoclast activity [55]. Osteogenic differentiation may be divided into three main stages, which are characterized by different levels of bALP: (1) proliferation (low bALP activity), (2) ECM synthesis (high bALP activity), and (3) ECM mineralization (moderate bALP activity) [43]. Since bALP is considered an early marker of osteogenic differentiation, in this study, its level was assessed in MC3T3-E1 cells after 8-day exposure to EO-derived compounds (Figure 2b). The performed experiments revealed that MC3T3-E1 cells produced higher amounts of bALP after exposure to carvacrol at the concentration of 37.50 µg/mL and α-pinene and β-pinene at the concentration of 300 µg/mL as compared to the control cells. The obtained results are consistent with the information found in the available literature, which reported a positive impact of α-pinene on bALP synthesis [22,56].

During the third phase of osteogenic differentiation, osteoblasts produce high levels of osteocalcin (OC) and osteopontin (OP), which are responsible for calcium binding and the proper process of ECM mineralization [43]. OC is a non-collagenous protein and is considered a late osteogenic differentiation marker [44]. In this study, a significant increase in OC synthesis in MC3T3-E1 cells was observed after 21 days of culture in the presence of 7.50 µg/mL cinnamaldehyde, 37.50 µg/mL carvacrol, 31.25 µg/mL (R)-(+)-limonene, and 300 µg/mL α-pinene (Figure 2c). Importantly, the stimulation of OC expression in osteoblasts after treatment with the α-pinene and limonene was also observed in other studies [12,15]. In turn, thymol at the concentration of 37.50 µg/mL and (S)-(−)-limonene at the concentration of 31.25 µg/mL significantly decreased the synthesis of OC.

### 3.4. Evaluation of ECM Mineralization

**Figure 3:**
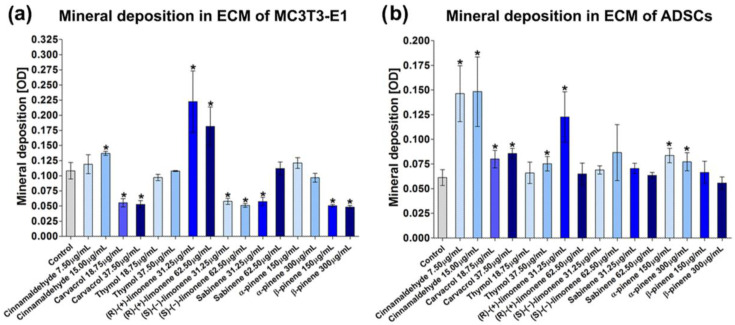
Quantitative evaluation of mineral deposition in ECM of (**a**) mouse primary calvarial preosteoblasts (MC3T3-E1 Subclone 4) and (**b**) canine adipose tissue-derived mesenchymal stem cells (ADSCs) assessed by Alizarin Red S staining. The results were expressed as OD at 450 nm (* statistically significant results compared to control, *n* = 3, *p* < 0.05, unpaired *t*-test).

**Figure 4:**
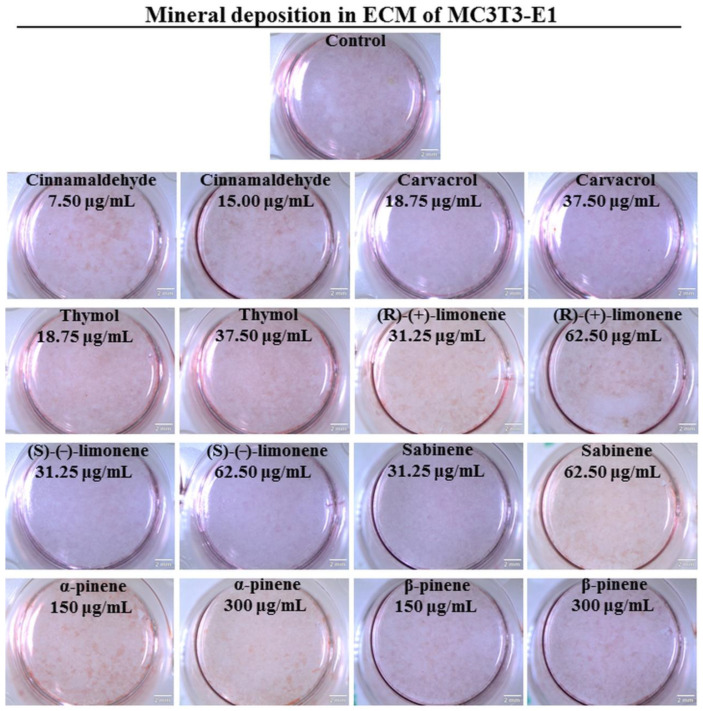
Qualitative evaluation of mineral deposition in the ECM of mouse primary calvarial preosteoblasts (MC3T3-E1 Subclone 4) assessed by Alizarin Red S staining and followed by stereoscopic microscope visualization (scale bar = 2 mm).

**Figure 5:**
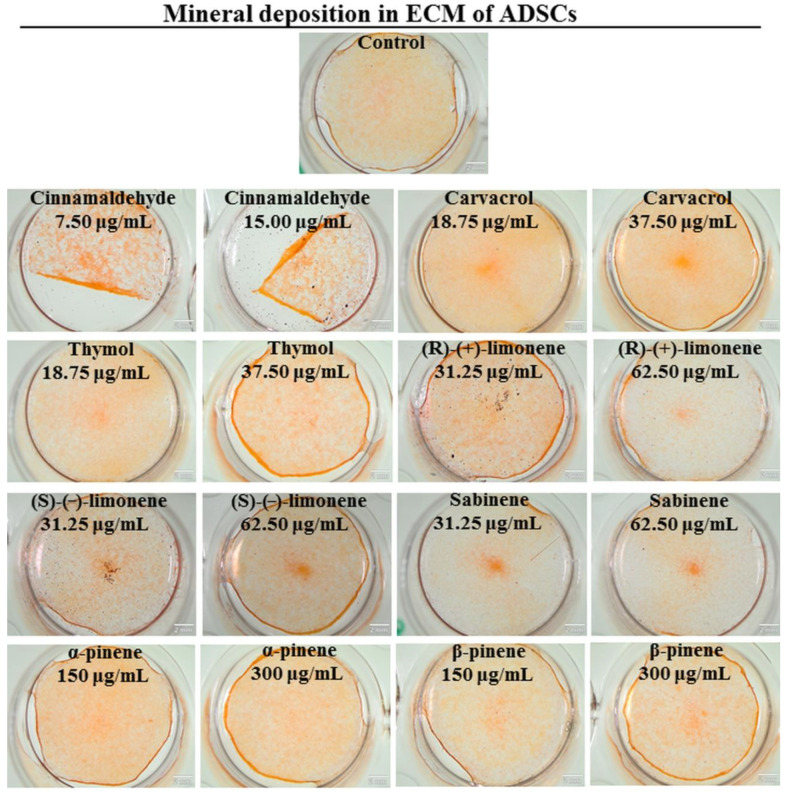
Qualitative evaluation of mineral deposition in the ECM of canine adipose tissue-derived mesenchymal stem cells (ADSCs) assessed by Alizarin Red S staining and followed by stereoscopic microscope visualization (scale bar = 2 mm).

An appropriately mineralized bone ECM is very important for the prevention of osteoporosis since it allows for the maintenance of sufficient bone strength and makes the bone resistant to fracture [57]. Thus, in the next step of the study, ECM mineralization was assessed using MC3T3-E1 preosteoblasts and ADSCs to confirm the obtained results. Quantitative and qualitative evaluations of mineral deposition in the ECM of MC3T3-E1 cells and ADSCs were performed (Figure 3, Figure 4 and Figure 5). In the case of MC3T3-E1 cells, a statistically significant increase in mineral synthesis was observed only after treatment with cinnamaldehyde at the concentration of 15.00 µg/mL and (R)-(+)-limonene at the concentrations of 31.25 and 62.50 µg/mL (Figure 3a). Carvacrol, (S)-(−)-limonene, β-pinene, as well as sabinene at the concentration of 31.25 µg/mL resulted in about a two-fold reduction in the mineral deposition in ECM. Stereoscopic microscope visualization of MC3T3-E1 culture subjected to the treatment with EO-derived compounds after Alizarin Red S staining confirmed quantitative data (Figure 4). Interestingly, unlike MC3T3-E1 cells, ADSCs showed significantly better ECM mineralization after treatment with EO-derived compounds (Figure 3b and Figure 5). Cinnamaldehyde, carvacrol, thymol (only at a concentration of 37.50 µg/mL), (R)-(+)-limonene (only at a concentration of 31.25 µg/mL), and α-pinene significantly stimulated ECM mineralization. Importantly, cinnamaldehyde caused an almost three-fold increase in the mineral deposition in the ECM of ADSCs compared to the control cells. Figure 5 shows images of well-visible mineral deposits in the ECM of ADSCs that were stained with Alizarin Red S solution. It is worth mentioning that in a previous study, it was proven that ADSCs are characterized by a high degree of ECM mineralization [58].

Osteogenic differentiation results in bone ECM formation and mineral deposition. ECM calcification is strictly related to type I collagen production, which serves as a framework for the process. Moreover, other proteins that have mineral binding properties, such as osteopontin and osteocalcin, participate in the regulation of the mineralization process [43]. In the conducted studies, no considerable correlation was observed between the production of Col I/OC and ECM mineralization, with the exception of the effect of cinnamaldehyde and (R)-(+)-limonene. Cinnamaldehyde at a concentration of 15.00 µg/mL increased Col I and OC synthesis by MC3T3-E1 cells, which had a direct positive effect on mineral deposition in the ECM of both MC3T3-E1 cells and ADSCs. Similarly, (R)-(+)-limonene at a concentration of 31.25 µg/mL increased OC production by MC3T3-E1 cells and significantly enhanced ECM calcification in MC3T3-E1 cells and ADSCs. However, it should be noted that the function of osteocalcin in the mineralization process is not fully understood. Studies have shown a correlation between increased expression of osteocalcin and high degrees of bone mineralization or formation [59,60], but the absence of OC did not interfere with mineralization in mice lacking the osteocalcin gene [61]. Moreover, there are also reports presenting an inhibitory effect of osteocalcin on the growth of hydroxyapatite crystals [62,63]. Recent research, however, suggests that osteocalcin may play a key role in the correct spatial arrangement of apatite crystals in relation to collagen fibers and affect bone strength [64].

Essential oils found in commonly consumed herbs, fruits, and spices may be candidates for preventing osteoporosis through diet. In vivo studies confirmed that the presence of herbs in the diet, which are rich in essential oils and monoterpens, inhibited bone resorption in a rat animal model [65]. The effect of rosemary and thyme essential oils on the prevention of osteoporosis was also studied using rats fed a diet low in calcium. In contrast to the positive control rats (low calcium diet), which had osteoporotic bone changes, no bone loss was observed in rats supplemented with rosemary and thyme powder. In addition, the presence of thyme and rosemary in the diet had a positive effect on the increase in the level of calcium and vitamin D3 in plasma, improvement of bone mineral density, and protection against oxidative stress [66]. In another study, Kania et al. demonstrated normalization of bone turnover markers (including C-telopeptide collagen type I and osteocalcin) in ovariectomized rats treated with *Cinnamomum burmanini* Blume extract [67]. Essential oils are a mixture of substances with a wide spectrum of biological activity. It is also important to select specific substances responsible for a given activity. Weng et al. showed that the combination of cinnamaldehyde with β-tricalcium phosphate (β-TCP) promoted osteogenesis and angiogenesis in bone defects caused by osteoporosis in rats [68]. Zheng et al. proved that bergapten can prevent LPS-induced osteoclast differentiation, bone resorption, and in vitro osteoclast survival [25]. Moreover, essential oils and monoterpenes can be immobilized as bioactive compounds in bone scaffolds supporting bone regeneration. Jaganathan et al. produced scaffolding material based on polyurethane, rosemary oil, and copper sulfate. The developed EO-enriched biomaterial had an optimal surface morphology and increased the viability of fibroblasts grown on its surface [69]. In turn, Mani et al., during in vitro studies, noted increased deposition of calcium compounds on the surface of a scaffold consisting of polyurethane, eucalyptus oil, and zinc nitrate compared to the biomaterial made of polyurethane alone [70].

## 4. Conclusions

The side effects of long-term use of conventional drugs have prompted scientists to search for compounds of natural origin that could be a safe alternative in the treatment of osteoporosis. In this research, MC3T3 cells were used as a model cell line to select the most promising EO-derived compounds that could potentially be used to enhance osteogenic differentiation and ECM mineralization. The ECM mineralization process, due to its key role in bone strength, has been additionally tested using adipose tissue-derived mesenchymal stem cells (ADSCs). Considering all performed tests, among the tested EO-derived compounds, cinnamaldehyde and (R)-(+)-limonene are the most likely to have a positive impact on bone ECM synthesis and mineralization. The mentioned compounds not only accelerated proliferation and OC synthesis by MC3T3-E1 preosteoblasts, but also significantly enhanced the ECM calcification of both preosteoblasts and mesenchymal stem cells. Thus, based on the research conducted, it can be assumed that cinnamaldehyde and (R)-(+)-limonene seem to be promising compounds that can be selected for further detailed research in order to confirm their biomedical potential in the chemoprevention or treatment of osteoporosis.

## Figures and Tables

**Table 1:** Occurrence and structural formulas of the tested EO-derived compounds.

EO-Derived Compounds	Structural Formula	Occurrence
**(R)-(+)-limonene**		*Citrus* spp., *Lippia* spp., *Artemisia* spp. [31]
**(S)-(−)-limonene**	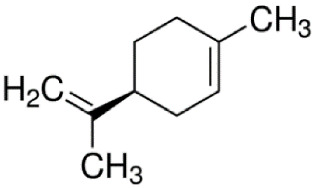	*Pinus* spp., *Mentha* spp. [31]
**sabinene**	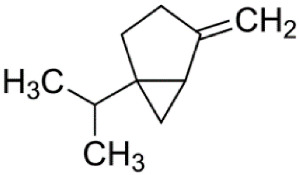	*Betula* spp. [32] *Citrus* spp. [33]; *Oregano* spp. [34]
**carvacrol**	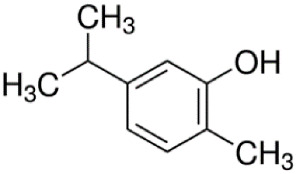	*Origanum* spp., *Thymus vulgaris*, *Lepidium flavum*, *Citrus aurantium* var. *bergamia Loisel* [35,36]
**α-pinene**	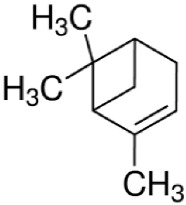	*Juniperus* spp., *Pinus* spp., *Piper nigrum*, *Cannabis sativa* L., *Achillea millefolium*, *Artemisia tridentata* [37,38]
**β-pinene**	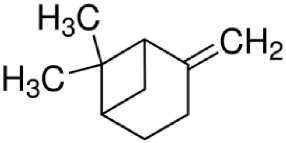	*Pinus* spp., *Cupressaceae* spp., *Artemisae* spp., *Citrus* spp. [37]
**thymol**	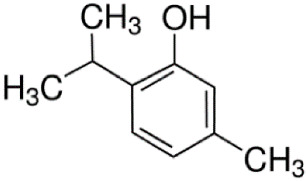	*Thymus* spp., *Satureja* spp., *Ocimum* spp., *Thymbra* spp., *Origanum* spp., *Monarda* spp., *Euphrasia rostkoviana Hayne*, *Nigella sativa* L., *Trachyspermum ammi* (L.) [39]
**cinnamaldehyde**	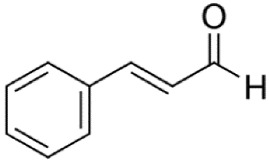	*Cinnamomum* spp. e.g., *Cinnamomum cassia*, *Cinnamomum verum* [40]

**Table 2:** Selected non-toxic concentrations of eight commercially available EO-derived compounds based on MTT cytotoxicity test.

Compound	Selected Non-Toxic Concentration [μg/mL]	Cell Viability [% of Negative Cytotoxicity Control] ± SD
cinnamaldehyde	7.50	102.9 ± 0.8
	15.00	101.5 ± 3.3
carvacrol	18.75	108.3 ± 1.3 *
	37.50	103.8 ± 1.9
thymol	18.75	103.7 ± 4.6
	37.50	105.6 ± 1.3
(*R*)-(+)-limonene	31.25	104.8 ± 3.2
	62.50	106.6 ± 4.3
(*S*)-(−)-limonene	31.25	110.2 ± 0.6 *
	62.50	107.6 ± 1.2 *
sabinene	31.25	96.3 ± 5.2
	62.50	99.8 ± 4.1
α-pinene	150	106.2 ± 0.6 *
	300	106.3 ± 2.3
β-pinene	150	104.6 ± 3.5
	300	102.9 ± 2.5

* statistically significant results as compared to negative cytotoxicity control (cells cultured in a medium without EO-derived compounds), *p* < 0.05, unpaired *t*-test.

## Data Availability

The raw/processed data required to reproduce these findings can be obtained from the corresponding author (agata.przekora@umlub.pl) upon reasonable request.
